# Error Assessment and Mitigation Methods in Transient Radar Method

**DOI:** 10.3390/s20010263

**Published:** 2020-01-03

**Authors:** Ali Pourkazemi, Salar Tayebi, Johan H. Stiens

**Affiliations:** Department of Electronics and Informatics, Vrije Universiteit Brussel, Pleinlaan 2, BE-1050 Brussels, Belgium; salar.tayebi@vub.be (S.T.); jstiens@etrovub.be (J.H.S.)

**Keywords:** blind method/algorithm, electromagnetic wave, geometric and electromagnetic characteristics, multilayer structures, sub-wavelength thickness measurements, non-destructive testing, non-metallic, lossy-lossy interface

## Abstract

Transient Radar Method (TRM) was recently proposed as a novel contact-free method for the characterization of multilayer dielectric structures including the geometric details. In this paper, we discuss and quantify the intrinsic and systematic errors of TRM. Also, solutions for mitigating these problems are elaborated extensively. The proposed solution for error correction will be applied to quantify experimentally the thickness of several single-layer dielectric structures with thicknesses varying from larger to smaller than the wavelength. We will show how the error correction method allows sub-wavelength thickness measurements around λ/5.

## 1. Introduction

It hardly needs any saying that microwave, millimeter, and THz waves are step-by-step penetrating the application fields of non-destructive testing (NDT). For several materials, this part of the electromagnetic spectrum seems superior to more traditional techniques such as ultrasound and X-ray [1,2,3,4]. The wide application domain of this spectrum in NDT has triggered the research community to find more versatile, faster, more accurate and more robust techniques. One of the latest techniques is called the Transient Radar Method, which features many of these advantageous properties with respect to competitive solutions existing today. Part of the research in this domain has been focusing on mathematical algorithms to increase the accuracy at the cost of more calculation power and processing time. A special challenge is still the combined retrieval of the complete geometric and electromagnetic characteristics of Multilayer Structures (MLS) in a completely blind way. Solutions proposed for this important challenge in NDT show one or more disadvantages, such as the time-consuming process, a need for higher frequency resolution, a need for larger frequencies up to the THz range, stability [5], increased bandwidth for creating ultra-narrow pulses in the time domain or sweeping frequency in spectroscopy, etc. [5,6,7]. In the following, some other limitations, especially in the THz range, will be mentioned. Firstly, THz time-of-flight has been proposed to be a strong inspection technique, particularly for a depth resolution of less than 0.1 mm; its drawbacks include system size, cost, and complexity, stemming from the use of femtosecond laser pulses and other components needed for data acquisition [8]. Secondly, Continuous Wave (CW) THz radar, using Frequency-Modulated CW (FMCW), has been applied [9,10]. Most applications based on the THz FMCW radar have been proposed in online imaging of concealed weapons under clothes at few-meter distances. Frequency sweeping range has the main role in the rate of depth resolution. Thirdly, optimization algorithms such as genetic algorithm and sequential quadratic programming are applied to S-parameter measurements to compute the permittivity of dielectric multi-layer structures in free-space. Both techniques are partly slow, but with reasonable accuracy [11]. Tomographic imaging is based on computed tomography (CT), which uses the pulse or the CW systems [12,13]. The next technique is synthetic aperture (SA) imaging, which is based on FMCW radar, which requires ultra-wide frequency range. Besides, this method requires prior knowledge of the material under test to increase its accuracy [14]. It typically uses millimeter-wave radiation but yields only a few millimeters depth and lateral resolution [15,16]. Finally, one of the most famous methods that has been used to determine the complex dielectric properties of materials from S-parameter measurements is known as the Nicolson-Ross-Weir (NRW) method [17,18]. This method requires ultra wideband, with high frequency resolution to extract relative permittivity or permeability values even for a very tiny single layer. Besides, retrieving the electromagnetic characteristics of a single layer is a big challenge for this algorithm [19,20].

Hence, finding new illumination methods and algorithm(s) that can cope with all mentioned problems is very appealing. In the next paragraph, we present the recently introduced method called the TRM [21,22,23]. In TRM, a multi-layer dielectric or magnetic structure is periodically illuminated with a monochromatic CW signal and the time-dependent reflection signal from the multi-layer structure under investigation is recorded. Each illumination period starts with a step function, featuring first-order low-pass filter characteristics with a finite rise time. This periodic step-wise illumination introduces a first transient phenomenon in the recorded reflection signal. The second transient phenomenon in the reflection signal is emerging from the time-dependent multiple reflections on all interfaces of the structure under investigation. The performance of TRM is determined by the time resolution of the measurement system instead of the frequency resolution. As time resolution is critical, superior performance can be obtained by the equivalent sampling principle such as in sampling oscilloscopes. TRM can handle thin samples as well as thick ones very well. TRM is fast, completely blind, and with good accuracy, without needing prior knowledge.

In the second section of this article, the basic data accumulation technique of TRM will be discussed. It will be illustrated by means of a two-layer system, comprising a first thin layer and a second semi-infinite layer to reduce the complexity of the reflection signal pattern. For simplicity, the rise time of the switch is assumed infinitesimally small. In the third section, the impact of finite rise time and dispersion of materials is quantitatively assessed by an appropriate mathematical model. In this model, the plane wave approximation is still used. In the fourth section of the paper, the data extraction procedure is applied to a single-layer structure to extract the geometric and electromagnetic characterization. Mitigation of intrinsic and systematic errors and experimental results are studied in sections five and six, respectively. Finally, the conclusions are drawn in the last section.

## 2. Measurement Methodology

**Figure 1:**
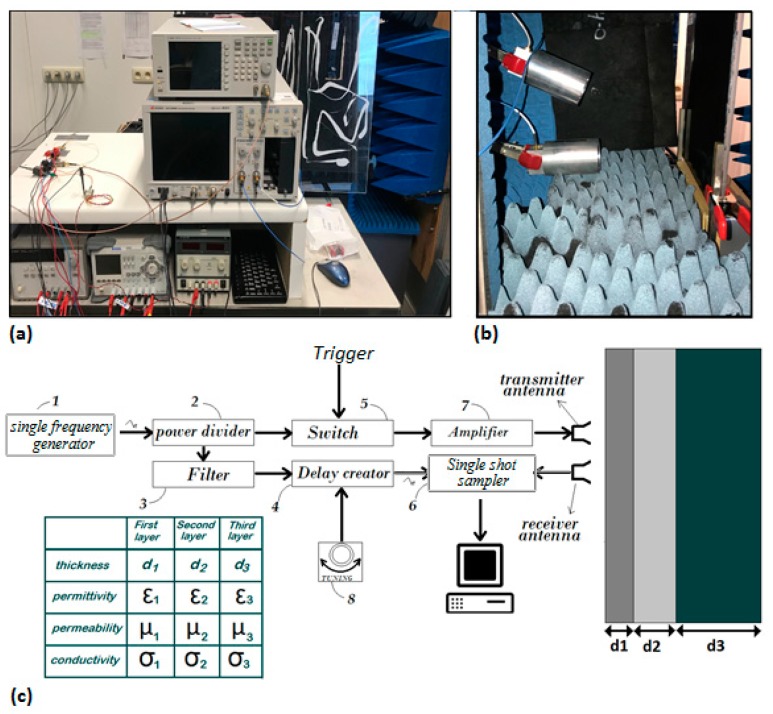
(**a**) The general set-up of TRM. (**b**) The transmitter and receiver antennas in the measurement set-up. (**c**) A block diagram explaining the set-up. Thickness, permittivity, permeability and conductivity of each layer can be measured by means of TRM.

**Figure 2:**
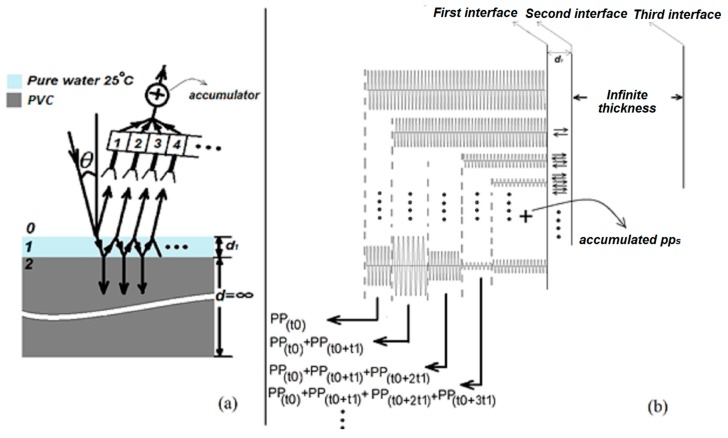
(**a**) Schematic illustration of the propagation multi-pathways of an EMW incident on a double-layer structure and partially bouncing forth and back between the various interfaces. (**b**) Schematic illustration of the time evolution of the EMW being reflected on the various interfaces of the double-layer structure with θ = 0. The nose of the illustrated reflected wave hits the detector after the round-trip time.

In TRM, the sample under test (SUT) in free space is periodically illuminated with a monochromatic electromagnetic continuous wave (EMCW) and the time-dependent reflected trace is captured by a narrow-band receiver (see Figure 1). As such, TRM can be categorized as a special kind of Time Domain Reflectometry (TDR) method without the need for ultra-short pulses. To simultaneously extract the thickness, complex permittivity and complex permeability of each layer, a dual-frequency illumination trace is needed [21,22,23]. Periodic illumination is obtained by on-off keying a Single Pole Single Throw (SPST) switch arranged between the CW generator and the SUT [22,23]. The finite rise time of the switch introduces a step function with first-order low pass characteristics. At the receiver side, the time-dependent voltage amplitude is acquired such as in a sampling oscilloscope. In other words, in each illumination cycle, one or more samples of the transient reflection signal with an infinitesimally small time resolution are acquired. To clarify the method, we describe the procedure for a two-layer system immersed in an air environment and illuminated by an electromagnetic wave (EMW) launched from the antenna, as illustrated in Figure 2a. A first thin layer consisting of water is placed on the second semi-infinite Polyvinyl Chloride (PVC) layer, which acts as a substrate layer. In this example, for illustration purposes only, we suppose that the conductivity and relative permeability for the two layers are zero and one, respectively. Any properties related to air, water, and PVC are denoted by subscript 0, 1 and 2. The thickness of the PVC layer is assumed semi-infinite in this illustration to avoid complex time-dependent reflection patterns. The angle of the incident wave is θ. In Figure 2, we also supposed that several receivers denoted by their enumeration number n = 1, 2, 3, 4 … individually receive reflection signals from unique propagation paths (*PP*) of the incident EM illumination signal. We also supposed that these *PP*s do not mix with each other.

When the antenna starts to illuminate the SUT, different reflection signals will be generated at different moments in time. At first, after a certain delay, the first receiver will start to receive the signal reflected on the front surface of the SUT, then the second receiver will start to receive reflection signals with a longer propagation path compared to the signal received by the first receiver and similar to what happened for the first and second receiver, can be seen for the rest of the receivers as well. When these receivers are accumulated in the accumulator device, at discrete moments in time, one extra receiver will contribute to the accumulator signal, leading to a discontinuity in amplitude and phase of the accumulated signal. In between these discontinuities, the accumulated signal looks like a CW signal. After sufficient time, the contribution of an extra receiver will be infinitesimally small, such that the accumulator leads to a continuous monochromatic wave signal. At that moment, the steady-state Fresnel reflection coefficient of the multi-layer structure is reached. If θ = 0, all *PP*s will be redirected along the surface normal and accumulated in a single receiver and will lead to the Transient Reflected Signal (TRS) as illustrated in Figure 2b. From here onward, we assume for all calculations that θ = 0. We assume that the emitted waves are plane waves such that reflection and transmission properties at each interface can be easily derived. It is obvious from Figure 2b that the receiver will catch the nose of the reflection trace after the Round-Trip Time (RTT) and will continue to receive traces as long as the transmitter antenna is illuminating the SUT [21,22,23]. At each interface, the EMW energy will be divided into 2 parts, including reflection and transmission. The transmitted trace will propagate towards the next boundary on its way, being absorbed within that layer depending on the material properties of the layer. At the next interface, an equivalent division of the EMW will happen. In such a way, a round-trip time and absorption in each layer are defined. The waves reflected at each boundary will gradually superpose when time evolves. In Figure 2a, we schematically show the reflected wave originating from the two-layer system when θ = 0. For better understanding, in Figure 2a, it is supposed that the switching time for the switch is zero.

## 3. Assessment of the Impact of Finite Rise Time and Material Dispersion on TRM Results

A first challenge that needs to be tackled is a quantitative derivation of the bandwidth associated with the TRM and consequently the impact of the associated dispersion phenomena within that bandwidth on the TRM results. The on-off keying of the switch controlling the illumination of the SUT leads to a periodic step function like an illumination pattern. The finite rise time of this switch introduces a transient in the illumination pattern. The simplest model for a switch with finite rise time is a first-order low pass filter system. In the time domain, the transfer characteristics of the switch are read as: $A_{\max}\left(1-e^{-\frac{t}{\tau_{RC}}}\right)$, where *A*_max_, *t*, and *τ*_RC_ refer to the maximum amplitude of the emitter, time (only for t > 0) and the RC-time constant of the switch, respectively. The rise time *τ*_r_ defined as the growth from 10% to 90% of the step pulse amplitude is then related to the RC time constant as *τ*_r_ = 2.197 *τ*_RC_. The frequency transform in the normalized form will read as:

(1) $$H_{s}\left(\omega \right)=\delta \left(\omega \right)-\frac{1}{1+j\omega \tau_{\mathrm{RC}}}.$$

Finally, the bandwidth (BW) of the first-order low pass filter model can be related to the rise time [24,25,26]:

(2) $$BW=\left(\frac{0.35}{\tau_{r}}\right).$$

This BW is located inside the baseband but as the switch modulates the carrier frequency, the required BW will be doubled. When one uses a switch with *τ*_r_ = 1 ns in the TRM set-up, one derives that at the receiver side one needs only a BW of 700 MHz [23]. For a carrier frequency of 10 or 70 GHz, this leads to a 7% and 1% relative BW, respectively. To assess the impact of this BW, the material dispersion characteristics of the MLS in such BW around the carrier frequency needs to be judged. To estimate the impact of the material dispersion, we take the two-layer structure of Figure 2a with pure water at 25 °C in the first layer as an example of a material with extreme dispersion around 10 GHz. The semi-infinite substrate consisting of PVC is assumed to be non-dispersive with *ε*_r_ = 3. The angle of the incident wave is θ=0.

For the propagation path $PP_{\left(t_{0}\right)}\left(t,\omega \right)$ which is received by detector number 1,

(3) $$H{\left(d_{0},\omega \right)}_{PP_{\left(t_{0}\right)}}=\Gamma_{01}\left(\omega \right)e^{-j\omega t_{0}}.$$

Delay can be shown by means of the Fourier transform formula $f\left(t-t_{0}\right)\overset{ℱ}{↔}F\left(\omega \right)e^{-j\omega t_{0}}$ and $t_{0}$ can be obtained as below:

Here we set the distance between antennas and SUT equal to $d_{0}$ which then also defines the RTT between transmitter and receiver $t_{0}=2\frac{d_{0}}{C}$, whereby c corresponds to the speed of light in vacuum. For the $PP_{\left(t_{0}+t_{1}\right)}\left(t,\omega \right)$ which is received by detector number 2,

(4) $$H{\left(d_{0},\omega \right)}_{PP_{\left(t_{0}+t_{1}\right)}}=T_{01}\left(\omega \right)T_{10}\left(\omega \right)\Gamma_{12}\left(\omega \right)e^{-j\omega t_{0}}e^{-j2k\left(\omega \right)d_{1}},.$$

where $d_{1}$ is the thickness of the water layer and k(ω) is equal to $\omega \sqrt{\mu_{0}\varepsilon_{0}}\sqrt{\varepsilon_{r}\left(\omega \right)}$. In Equation (4), we used the Green function for dispersive materials as follows [27,28,29].

(5) $$H\left(z,\omega \right)=e^{-jk\left(\omega \right)z}$$

where ω, k(ω) and z refer to angular frequency, frequency-dependent wave number due to material dispersion and propagation distance, respectively.

For the $PP_{\left(t_{0}+2t_{1}\right)}\left(t,\omega_{0}\right)$ which is received by detector number 3,

(6) $$H{\left(d_{0},\omega \right)}_{PP_{\left(t_{0}+2t_{1}\right)}}=T_{01}\left(\omega \right)T_{10}\left(\omega \right)\Gamma_{10}\left(\omega \right)\Gamma_{12}^{2}\left(\omega \right)e^{-j\omega t_{0}}e^{-j4k\left(\omega \right)d_{1}}.$$

For the $PP_{\left(t_{0}+3t_{1}\right)}\left(t,\omega_{0}\right)$ which is received by detector number 4,

(7) $$H{\left(d_{0},\omega \right)}_{PP_{\left(t_{0}+3t_{1}\right)}}=T_{01}\left(\omega \right)T_{10}\left(\omega \right)\Gamma_{10}^{2}\left(\omega \right)\Gamma_{12}^{3}\left(\omega \right)e^{-j\omega t_{0}}e^{-j6k\left(\omega \right)d_{1}}.$$

In Equations (3)–(7), $t_{1},\;T_{mn}\left(\omega \right)$ and $\Gamma_{mn}\left(\omega \right)$ refer to the RTT due to signal front delay (explained in next paragraph), the transmission coefficient, and the reflection coefficient with the subscript indices determined by the two layers constituting the interface.

In addition, if f(t) and $h_{s}\left(t\right)$ are defined as single tone and non-ideal step function with given rise time, the time domain multiplication of them results in the frequency domain convolution of both. In this case, by means of $\left(f\left(t\right)\times h_{s}\left(t\right)\right)*h\left(t\right)\overset{ℱ}{↔}\left(F\left(\omega \right)*H_{s}\left(\omega \right)\right)\times H\left(\omega \right)$, we have:

(8) $$PP_{\left(t_{0}+nt_{1}\right)}\left(t,\omega \right)=\frac{1}{2\pi }\int_{-\infty }^{\infty }R_{n}\left(\omega \right)e^{j\omega t}d\omega ,n=0,1,2,\ldots ,$$

(9) $$R_{n}\left(\omega \right)=\left(F\left(\omega \right)*H_{s}\left(\omega \right)\right)\times H{\left(d_{0},\omega \right)}_{PP_{\left(t_{0}+nt_{1}\right)}}$$

$R_{n}\left(\omega \right)$ is produced by the convolution (*) and the multiplication (×) of three functions as follows:

(10) $$F\left(\omega \right)=ℱ\left(sin\left(\omega_{0}t\right)\right)=\frac{\pi }{j}\left[\delta \left(\omega -\omega_{0}\right)-\delta \left(\omega +\omega_{0}\right)\right].$$

F(ω), is the Fourier transforming of a sinus curve at a carrier frequency ω_0_.

$H_{s}\left(\omega \right)$ is a simple transfer function model of the switch with its corresponding rise time. On the other hand, $F\left(\omega \right)*H_{s}\left(\omega \right)$ describes the incident trace.

$H{\left(d_{0},\omega \right)}_{PP_{\left(t_{0}+nt_{1}\right)}}$ describes the frequency response for any individual *PP* as follows:

(11) $$\{\begin{array}{c} H{\left(d_{0},\omega \right)}_{PP_{\left(t_{0}\right)}}=\Gamma_{01}\left(\omega \right)e^{-j\omega t_{0}},n=0\\ H{\left(d_{0},\omega \right)}_{PP_{\left(t_{0}+nt_{1}\right)}}=T_{01}\left(\omega \right)T_{10}\left(\omega \right)\Gamma_{10}^{n-1}\left(\omega \right)\Gamma_{12}^{n}\left(\omega \right)e^{-j\omega t_{0}}\\ e^{-j2nk\left(\omega \right)d_{1}},n>0 \end{array}$$

Finally, as we mentioned before, θ=0, the TRS from a single-layer dispersive material which is placed on top of the infinitely thick non-dispersive and non-metallic layer can be written as follows:

(12) $$TRS(t,\omega_{0})=\sum_{n=0}^{n=\infty }PP_{\left(t_{0}+nt_{1}\right)}\left(t,\omega \right).$$

In simulation, we take the **Front Velocity** and **Causality** into account. Moreover, if this matter is dispersive too, the whole dispersed harmonics also satisfy causality. In the considered bandwidth, harmonics with the largest propagation speed will arrive sooner than others. The signal-front delay is generally defined as follows:

(13) $$t_{\mathrm{signal}-\mathrm{front}\;\mathrm{delay}}=\underset{\omega \to \infty }{\lim}\left(\frac{\varphi \left(\omega \right)}{\omega }\right).$$

where φ(ω) is the phase as a function of angular frequency. For the considered bandwidth, it can be rewritten as follows:

(14) $$t_{\mathrm{signal}-\mathrm{front}\;\mathrm{delay}}=\frac{d}{c_{\max}\left(\omega \right)}.$$

where d and $c_{\max}\left(\omega \right)$ refer to the distance and maximum propagation speed for a certain harmonic that propagates in a special dispersive matter. Hence, in Equations (4)–(7), $t_{1}$ refers to $t_{\mathrm{signal}-\mathrm{front}\;\mathrm{delay}}$.

**Figure 3:**
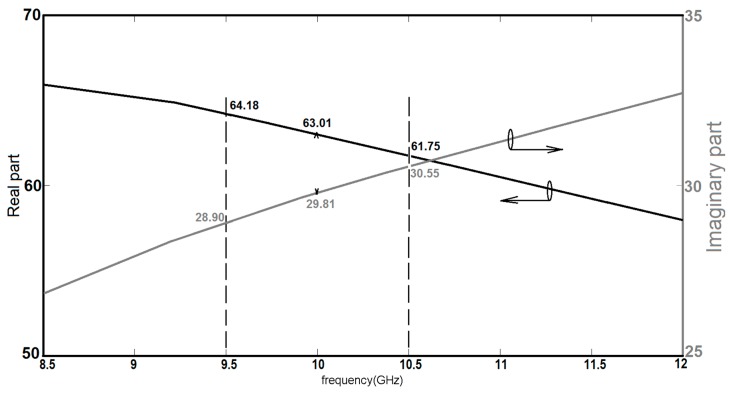
The real and imaginary part of permittivity for pure water at 25 °C.

To assess the impact of material dispersion on the TRM signal, water as a most dispersive material was selected. In fact, water around 10 GHz is a worst-case scenario. In the considered simulation, a 1 GHz bandwidth instead of 700 MHz was assumed for the frequency response of the switch. In this simulation, 5 complex permittivity values of pure water at 25 °C for 1 GHz bandwidth around 10 GHz were derived from the measured data of Table 2 in [30]. By means of interpolation, more than 100.000 frequency-dependent permittivity points were created between 9.5 and 10.5 GHz (see Figure 3). The goal of the simulation was to investigate the impact of material dispersion on transient radar response without considering the noise floor and any other non-idealities. Simulations indicate; however, that the data points of $PP_{\left(t_{0}+nt_{1}\right)}\left(t,\omega_{0}\right)$ for n≥2,… are already below the noise floor.

Simulations were done for two different situations:

1. Non-dispersive material characteristics
2. Fully dispersive material characteristics

At first, to get validation, simulations have been executed for fully non-dispersive cases at three different frequencies: $\varepsilon_{r}\left(9.5\;\mathrm{GHz}\right)$, $\varepsilon_{r}\left(10.5\;\mathrm{GHz}\right)$ and $\varepsilon_{r}\left(10\;\mathrm{GHz}\right)$. These simulations results were compared with the fully dispersive case. As expected, the number of errors due to subtraction of fully dispersive trace from the non-dispersive traces for $\varepsilon_{r}\left(9.5\;\mathrm{GHz}\right)$ and for $\varepsilon_{r}\left(10.5\;\mathrm{GHz}\right)$ were larger than the number of error due to subtraction of fully dispersive trace from the non-dispersive trace for $\varepsilon_{r}\left(10\;\mathrm{GHz}\right)$.

**Figure 4:**
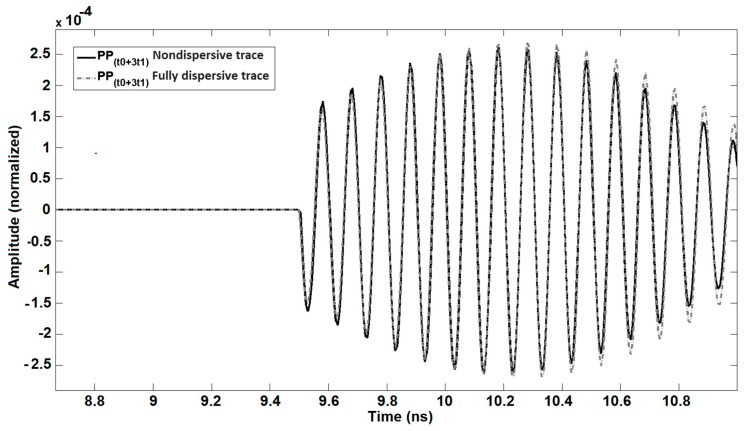
Simulation of nondispersive trace in 10 GHz and fully dispersive trace for $PP_{\left(t_{0}+3t_{1}\right)}\left(t,\omega_{0}\right)$. The amplitude is normalized.

In fact, the non-dispersive trace can be rebuilt by means of the “Ref” trace (it is explained further) for 10 GHz by applying phase delay and attenuation without taking into account the dispersion. Regarding our estimation, in the real measurement for this special case, we can only record up to $PP_{\left(t_{0}+t_{1}\right)}$ as the subsequent propagation paths due to the water absorption are below the noise floor for a 3-mm thick pure water layer at 25 °C. If we decrease the thickness, the probability for the detection of other PPs will be increased. PPs were simulated for a non-dispersive situation whereby permittivity of water was fixed at its value for *f* = 10 GHz and for a fully dispersive case considering a 1 GHz bandwidth around the central frequency of 10 GHz. The amplitude of $PP_{\left(t_{0}\right)}$ which is reflected from the surface of the water is comparable with the “Ref” trace. Regarding the simulations, the rise time related to the envelope function was changed due to phase jumps and dispersion. Also, there is a comparison between the non-dispersive and fully dispersive trace at 10 GHz as a frequency center. For the worst-case scenario, this comparison is considered for $PP_{\left(t_{0}+3t_{1}\right)}\left(t,\omega_{0}\right)$, since the longest wave propagation path is around 18 mm in water (See Figure 4).

The average of the relative error from $PP_{\left(t_{0}\right)}\left(t,\omega_{0}\right)$ up to $PP_{\left(t_{0}+3t_{1}\right)}\left(t,\omega_{0}\right)$ is less than 0.01%, 0.5%, 0.9%and 1.3%, respectively. These amounts of errors will be drastically smaller for weak or almost non-dispersive materials. Additionally, the impact of dispersion is strongly correlated with the layer thickness. It means, for the thinner layers, the small amount of error will be seen. Even for dispersive materials, it might be possible to reduce the impact of dispersion by shifting the frequency of the illumination to regions where the material dispersion is weaker. For instance, the material dispersion for water at 110 GHz is much smaller than the dispersion at 10 GHz [30]. Definitely, for many other materials such as PVC, different kinds of plastics, ceramics, and composites the residual material dispersion will be negligible for few millimeter thin layers and limited bandwidth associated with the switch. Thus, regarding the simulation results, we can state that dispersion is a challenge only if the thickness of the SUT is large but, for a lot of real samples the thickness is few millimeters at most.

## 4. Data Extraction Process

**Figure 5:**
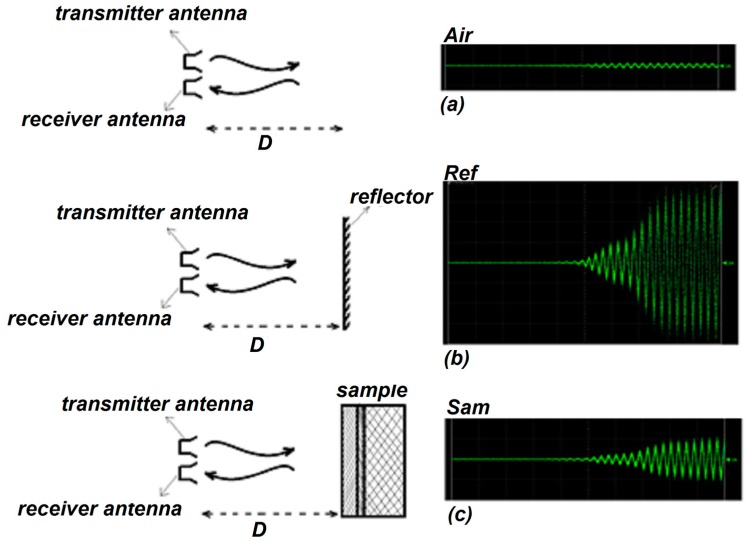
(**a**) Reflection due to crosstalk between transmitter and receiver antennas yields the “Air” signal. (**b**) Reflection from a perfect smooth metallic reflector placed in front of the antennas yields the “Ref” trace. (**c**) Reflection from multi-layered structure placed at the same location that reflector was placed, yields the “Sam” signal.

**Figure 6:**
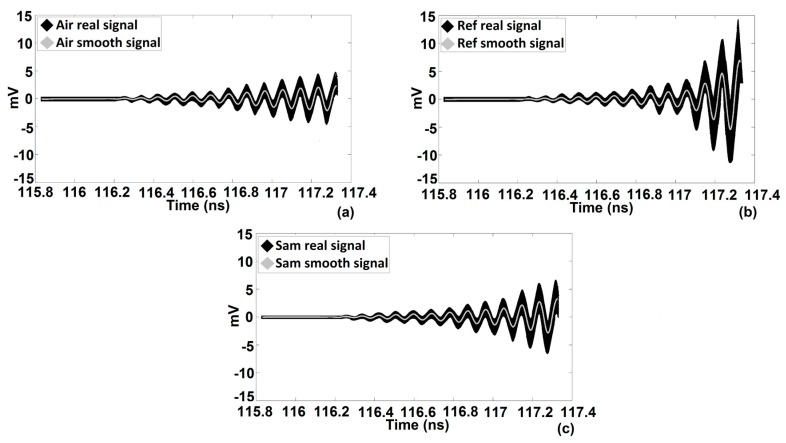
The real and smooth signals of (**a**) Air, (**b**) Ref and (**c**) Sam. Air is the trace due to crosstalk between the transmitter and receiver antennas, Ref is the trace generated from the PSMR and Sam is the trace generated from SUT.

In this section, we explain the procedure that was used to extract the thickness and complex permittivity of a single-layer structure by means of TRM. After recording the calibration [23] and the SUT traces, a histogram technique [31] is implemented to smooth the raw data (see the gray traces in Figure 6a–c). Moreover, calibration consists of two steps, which are named “Air” and “Ref” respectively. Air refers to the trace that is generated due to crosstalk of transmitter and receiver antennas, once neither the sample nor the Perfect Smooth Metallic Reflector (PSMR) are in front of the antennas. Ref refers to the trace that is generated due to a PSMR being in front of antennas instead of SUT. On the other hand, Sam is the trace generated from the SUT being in front of the transmitter and the receiver antennas (see Figure 5).

**Figure 7:**
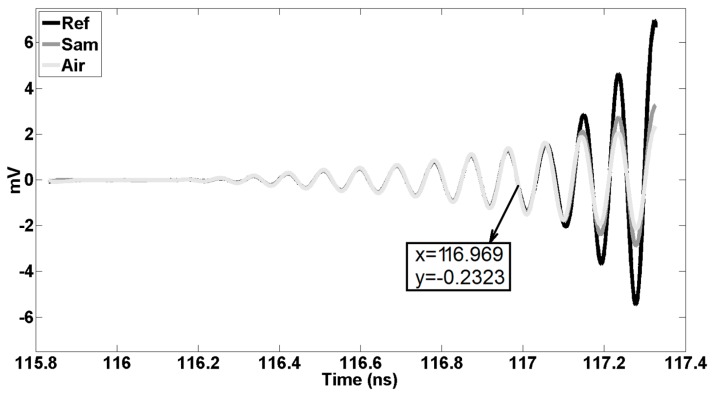
The first estimation of the nose, the growing deviation between the two curves.

In the next step, by means of reconciling Air and Ref traces (calibration data), the nose region will be predictable. Nose refers to the specific moment that Air and Ref start to deviate from each other. For instance, the difference between the Ref and Air traces at a certain time point is less than 100 μV; after a few tens of picoseconds, deviation becomes larger and larger. For a highly accurate determination of the nose, it is important to start comparing the Ref and Air traces well before the real nose trace pops up. For example, the first estimation of the nose is 116.969 ns (see Figure 6 and Figure 7). Real nose, obtained by the algorithm, happens at $t_{0}=$ 117.0045 ns, and we can use this nose point for all consecutive measurements if the position and distance of the sample do not change and if drift is zero, otherwise measurements should be repeated.

**Figure 8:**
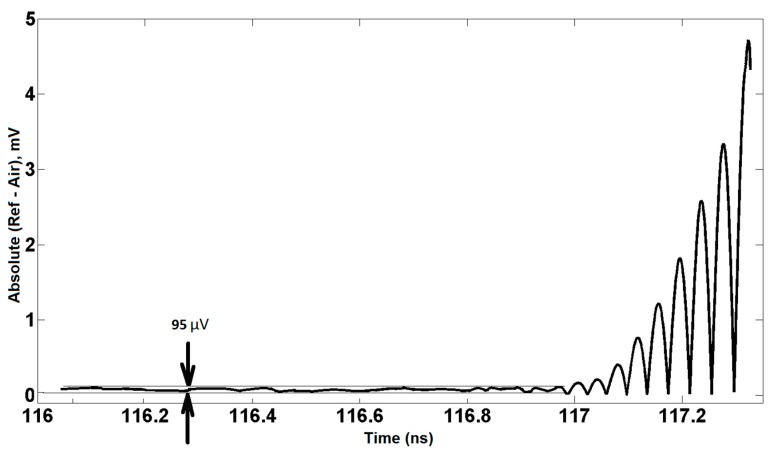
The real nose coinciding with the deviation between the two curves.

Additionally, for a more accurate determination of the nose, one can change the position of PSMR by moving it slightly towards the front and back (for more details, see calibration in [23]). Precision can be enhanced by making use of interpolation and a more precise resolution. For a homogenous single-layer structure, the set of traces is limited: $PP_{\left(t_{0}\right)}\;\mathrm{and}\;PP_{\left(t_{0}+nt_{1}\right)},n=1,2,3,\ldots$. After subtraction of the Air trace from the Sam [23] and Ref [23] traces, one can start to superpose the first parts of the ‘Sam-Air’ with ‘Ref-Air’ traces corrected with the appropriate attenuation and phase shift correction factors. It is worth mentioning that one can apply a phase shift to the trace which is not a pure sinusoidal curve by converting the trace from the time to the frequency domain, using the Fast Fourier Transform operator. After applying a correct phase shift to all the harmonics, one can return to the time-domain by means of the Inverse Fast Fourier Transform operator. After obtaining $PP_{\left(t_{0}\right)}$ and subtracting it from ’Sam-Air‘, the tracking of $PP_{\left(t_{0}+t_{1}\right)}$ will be started. The only difference between the reconstruction of $PP_{\left(t_{0}\right)}$ and $PP_{\left(t_{0}+t_{1}\right)}$ is using the delay parameter as well as the attenuation and phase shift parameters to obtain $PP_{\left(t_{0}+t_{1}\right)}$. Once $PP_{\left(t_{0}\right)}$ and $PP_{\left(t_{0}+t_{1}\right)}$ have been determined, the complex permittivity and thickness of the single-layer can be extracted. Calculation and reconstruction of other PPs such as $PP_{\left(t_{0}+2t_{1}\right)}$ can enhance the accuracy. However, reconstructing the Sam trace using all obtained PPs simultaneously can decrease the total error. PPs formulas and extraction data are explained in [22] and [23] extensively. Figure 6, Figure 7 and Figure 8 are traces obtained for a single layer of a Vubonite [32] slab with dimensions of 300 mm×300 mm×5.84±0.03 mm. Data from other samples that are given in Table 1 were obtained by applying the same algorithm but, only the results are reported.

For studying Table 1, the following definitions should be considered:

A1 denotes the mitigation of the error due to the angle of incidence.
A0 denotes the non-mitigation of the error due to the angle of incidence.
D1 denotes the mitigation of the error due to divergence.
D0 denotes the non-mitigation of the error due to divergence.

## 5. Mitigation of Intrinsic and Systematic Errors

Regarding the schematics of the transient radar set-up in [23], ideally, the SPST switch does not induce any losses, has a zero rise-time and does not feature any jitter. This means that at each trigger moment inducing the conductive state of the switch, the trace will abruptly start to propagate with a monochromatic amplitude in a perfectly repeatable way. In the ideal model, we also suppose that all the components are free from jitter, noise, and drift. Additionally, the ideal model also assumes a plane wave approximation, meaning that the emitted EMW does not diverge and, features planar phase fronts. In practice, all these idealities do not exist. To cope with jitter and noise as intrinsic errors, measurement repetition is useful but, it causes drift. Therefore, we need to compromise between accuracy (number of points) and drift. Divergence of the beam due to nonlinearity (systematic error) was considered during the calibration by means of a PSMR as a reference, slid along the propagation direction [21,22,23]. Another systematic error that must be taken into account is the angle of the two antennas [7].

### 5.1. Beam Divergence

**Figure 9:**
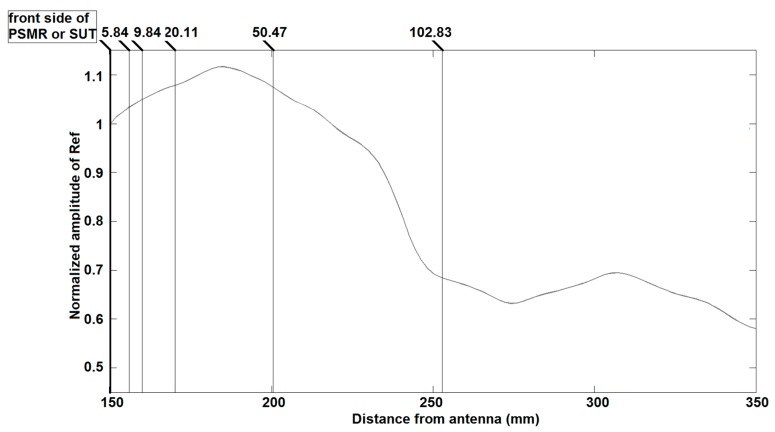
Normalized amplitude of Ref signal versus antenna distance.

The foundation for the extraction of data (layer thickness and dielectric permittivity) from TRM is based on the exact knowledge of the *PP*s. Although expressions exist to predict the distance-dependent power density along the propagation axis in the far-field (classic beam divergence) and even in the near-field (very nonlinear) for Gaussian or spherical beams, in this work, the distance-dependent power density was experimentally recorded every 0.1 mm, starting from the position of the SUT up to a distance at least four times the electrical thickness of the SUT (see Figure 9). In this way, a reference table quantifying the propagation divergence is created. It is clear that accuracy is improved by decreasing the sliding steps. The sliding back away of PSMR from the transmitter and receiver antennas induces a delay and attenuation that can be easily observed on an oscilloscope. As such, for the validation of the beam divergence compensation algorithm, the trace recorded for the PSMR located at 40 cm from the antennas can be rebuilt by Ref trace, obtained when the PSMR is located at only 15 cm by applying the appropriate delay and attenuation factors on the Ref trace that is recorded from PSMR at 15 cm. This means that the phase of Ref trace is not changed by the sliding, as was experimentally confirmed in [23] where we used a unique A as a constant amplitude for all PPs. The pattern of the power density for the monochromatic case and for isotropic materials has not been changed, but it will be compressed as a result of any refractive index n > 1. In order to mitigate the error due to divergence and the angle of two antennas, one should calculate the physical path that the wave propagates in the MLS. Then, based on this physical path, the amplitude will be determined based on Figure 9. For instance, amplitude coefficients of the $PP_{\left(t_{0}+t_{1}\right)}$, $PP_{\left(t_{0}+t_{1}+t_{2}\right)}$ and $PP_{\left(t_{0}+t_{1}+2t_{2}\right)}$ can be calculated as follows:

(15) $$A_{\left(t_{0}+t_{1}\right)}=A_{PSMR}\left(x_{1}\right),$$

(16) $$A_{\left(t_{0}+t_{1}+t_{2}\right)}=A_{PSMR}\left(x_{2}\right),$$

(17) $$A_{\left(t_{0}+t_{1}+2t_{2}\right)}=A_{PSMR}\left(2x_{2}-x_{1}\right),$$

where $A_{\mathrm{PSMR}}$, $x_{0}$, $x_{1}$ and $x_{2}$ refer to the position-dependent amplitude of the Ref trace, the coordinate of the front side of the PSMR or SUT, the coordinate of the interface between first and second layers, and the coordinate of the interface between second and third layers.

Hence the expression

(18) $$PP_{\left(t_{0}+t_{1}\right)}\left(t,\omega_{0}\right)=Ae^{-j2\beta_{0}d_{0}\;}e^{j\omega_{0}t}T_{01}T_{10}e^{\left(-2\alpha_{1}d_{1}\right)}e^{-j2\beta_{1}d_{1}\;}\Gamma_{12}U\left(t-t_{0}-t_{1}\right).$$

Should be replaced by

(19) $$PP_{\left(t_{0}+t_{1}\right)}\left(t,\omega_{0}\right)=A_{PSMR}\left(x_{1}\right)e^{-j2\beta_{0}d_{0}}\;e^{j\omega_{0}t}T_{01}T_{10}e^{\left(-2\alpha_{1}d_{1}\right)}e^{-j2\beta_{1}d_{1}}\;\Gamma_{12}U\left(t-t_{0}-t_{1}\right).$$

where $\beta_{0}$ and $\beta_{1}$ are defined as propagation constant in free space and propagation constant inside the SUT.

It is worth mentioning that this error correction cannot be applied to the extraction of parameters (permittivity neither the thickness) of the first layer. For the calculation of permittivity and thickness in the second layer, we should take the divergence into account as a systematic error. Additionally, the distance between the front side of SUT and the antennas can lead to the strengthening or weakening of this kind of error. For the experimental validation, it is most appropriate to use a single-layer homogeneous structure as the SUT. Finally, the best indicators for the evaluation of precision and error analyses of TRM measurements are the thickness of SUT and the relative permittivity of air behind the SUT. The first one can be measured by a caliper and the second one is $\varepsilon_{r}\left(\mathrm{air}\right)=1$.

### 5.2. The Angle of Two Antennas

The angle of the two antennas can create two kinds of systematic errors, as follows:

#### 5.2.1. Systematic Error Due to an Increase in the EM Wave Propagation Time in Each Layer Due to the Angle between the Two Antennas

**Figure 10:**
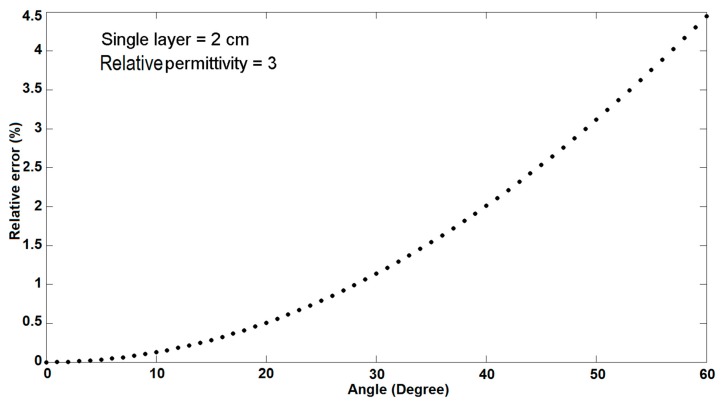
Relative error versus the angle between the two antennas for a 2 cm thin single-layer structure with relative permittivity equal to 3.

David J. Cook and his colleagues described the systematic error introduced due to the angle between transmitter and receiver antennas [7]. The subsequent dependence of the ratio between the real and experimental thickness on the angle between the antennas can be derived by Equation (20).

(20) $$D_{m}=\frac{2D_{T}}{cos\left(\frac{\beta }{2}\right)}=\frac{2D_{T}}{\sqrt{1-{\left(\frac{n_{0}}{n_{1}}sin\left(\frac{\alpha }{2}\right)\right)}^{2}}}.$$

where $D_{m},\;D_{T}$, $n_{1},$ $n_{0}$, *α* and *β* refer to the measured thickness of the first layer from the multilayer structure, the theoretical thickness, the refractive index for the first layer, the refractive index for the air, the angle of antennas and the angle between incidence and reflected wave in the first layer, respectively. For the sake of clarity, a simple simulation was executed for a 2 cm thin single-layer with $\varepsilon_{r}=3$ (see Figure 10). In this example, the angle between the two antennas was changed from zero to 60 degrees. This error can be strengthened or weakened in the subsequent layers. However, this depends on the permittivity of two layers (Snell’s law). Certainly, for the first layer, it will be weakened. The angle of transmitter and receiver antennas in the set-up was equal to 15 degrees, leading to a relative error of smaller than 0.5% in the first layer if $\varepsilon_{r}=3$.

#### 5.2.2. Systematic Error Due to Changes in the Permittivity by Means of the Angle of Two Antennas

Based on Fresnel’s equation, reflection coefficient is a function of electromagnetic parameters at the sides of the interface as well as the angle of incidence. Based on the electromagnetic boundary condition as well as Snell’s law, one can write (21), which is appropriate for a lossy-lossy interface while assuming that $\mu_{r}=1$ for two sides of the interface [29,33,34].

(21) $$\Gamma_{\left|\right|}=\frac{\sqrt{\varepsilon_{l}}\;{\left(\varepsilon_{r}-\varepsilon_{l}sin{\left(\theta_{i}\right)}^{2}\right)}^{0.5}-\varepsilon_{r}cos\left(\theta_{i}\right)}{\sqrt{\varepsilon_{l}}\;{\left(\varepsilon_{r}-\varepsilon_{l}sin{\left(\theta_{i}\right)}^{2}\right)}^{0.5}+\varepsilon_{r}cos\left(\theta_{i}\right)}.$$

where $\Gamma_{\left|\right|},\;\varepsilon_{l},\varepsilon_{r}$ and $\theta_{i}$ refer to the reflection coefficient at the interface for parallel polarization, the relative permittivity of the left-hand side (where the EMW is incident), the relative permittivity of the right-hand side of the interface and the angle of incidence, respectively. In (21), $\Gamma_{\left|\right|},\;\varepsilon_{l},\varepsilon_{r}$ and $\theta_{i}$ can be complex- or real-valued functions.

## 6. Experimental Results

The experimental results obtained for several PVC and Vubonite [32] sheets with different thicknesses are presented in Table 1. In this set-up, the angle between transmitter and receiver antennas is 15° ± 0.1°. These experimental data show that TRM is applicable for determining the complex permittivity of layers as well as the thicknesses of sheets which are approximately equal, larger or smaller than the wavelength. In addition, the mitigation of systematic errors was done step by step and the complex permittivity for air behind the single-layer structure was assessed before and after the error mitigation (see Table 1). The measurement process for each SUT was repeated more than three times. Regarding Table 1, the theoretical thickness of single-layer structures varies from less than 6 mm up to larger than 10 cm. For each sample, the complex permittivity and thickness were blindly obtained by TRM. Data without and with systematic error corrections are given in Table 1. To summarize the measurement and calculation processes of data in Table 1, we should pay attention to the following steps:

1. Measuring Ref, Sam, and Air signals.
2. Executing the sliding action of the PSMR backward from transmitter and receiver antennas and recording the data.
3. Finding the nose and extracting $PP_{\left(t_{0}\right)}\left(t,\omega_{0}\right)$ and $PP_{\left(t_{0}+t_{1}\right)}\left(t,\omega_{0}\right)$, respectively.
4. Calculating $\Gamma_{\left|\right|}$ front side and then relative permittivity of SUT and $t_{1}$.
5. Relative permittivity can be modified numerically by means of (21), $\Gamma_{\left|\right|}$ and angle of incidence. (mitigating error due to angle).
6. Modification of $\Gamma_{\left|\right|}$ backside based on sliding data. This leads to modified relative permittivity of air in the backside (mitigating divergence error).
7. Calculating thickness based on relative permittivity and $t_{1}$.

For studying Table 1, the following definitions should be considered:

- A1 denotes the mitigation of the error due to the angle of incidence.
- A0 denotes the non-mitigation of the error due to the angle of incidence.
- D1 denotes the mitigation of error due to divergence.
- D0 denotes the non-mitigation of error due to divergence.

Regarding the experimental results summarized in Table 1, errors related to the angle between the two antennas can only influence the calculations of permittivity of SUT. However, for extracting permittivity of air behind the sample, both error sources should be considered. The error due to the beam divergence is notable for thick layers while it is negligible for relatively thin layers. The relative error of the layer thickness is about 8% in the worst-case scenario, whereas in the best case, the error is less than 0.5%. Additionally, the relative error for the relative permittivity of air in the worst-case scenario is about 12%, whereas in the best case is less than 0.5%. For the two cases of PVC (20.11 mm) and PVC (9.84 mm), one can observe that the error of the air permittivity is less than 0.5% while the relative errors for their thicknesses are 8% and 2%, respectively.

Regarding the electromagnetic properties measured by TRM, the results can be seen in Table 1 but, to have a comparison with previously published data, we discuss results from another paper as well. In one study, by Riddle et al. [35], the permittivity of common plastics was measured as a function of temperature. The authors of this study used dielectric resonator method to extract dielectric constant of common plastics including PVC, and found the permittivity of PVC to be 2.70 at a frequency of 11 GHz and the temperature of 25 °C. Generally, it should be noted that the electromagnetic properties of materials depend highly on temperature as well as frequency during measurement. However, even with this high sensitivity to temperature and frequency, the achieved results by TRM in this study are in a good agreement with the study of Riddle et al. [35].

Actually, it means that the error sources can neutralize each other or at least the error due to the calculation of permittivity does not influence the thickness calculation dramatically. Hence, if one can extract the permittivity value with a limited relative error, one can expect that one can calculate the thickness with a reasonable error as well. However, further mitigation of the errors can be implemented by taking into account the bow of sample surfaces, intrinsic errors such as drift, non-perpendicularity of the sample under test versus beam direction, etc.

## 7. Conclusions

In this paper, the basic concepts of TRM were briefly repeated. The modality for the extraction of geometric and electromagnetic characteristics of each layer in an MLS by means of TRM was discussed. The required experimental bandwidth for TRM was analyzed. The measurement system used operating at 10 GHz did not induce more than 0.7 GHz bandwidth extension. The impact of dispersion on TRM was theoretically modeled and numerically investigated by executing a simulation of a single pure water layer covering an infinite thick PVC layer. The numerical analysis for water, one of the most dispersive materials at 10 GHz, showed that dispersion effect for thin material layers does not induce more than 1% error for layers with an equivalent thickness of less than 1 cm. Systematic errors such as divergent beam and angle of transmitter and receiver antennas were discussed extensively. It was provisionally shown that TRM can be more accurate when error correction is implemented to this method/algorithm. For example, the calculated thickness of the 102.83 ± 0.04-cm-thick PVC was 104.04 cm before error correction and 103.00 cm after error correction.

An overview of dielectric properties measurement techniques is written by Venskatesh et al. [36]. Although the accuracy defined for each method in this study [36] is not quantified, it is enough to show that the accuracy of TRM, after error mitigation would be at high level even in the worst-case scenario.

Finally, Table 1 summarizes all experimental data before and after the error corrections. It is demonstrated that the fully blind TRM method derives the thickness values typically with an error of less than 4%, exceptionally 8%; and for the permittivity of air e.g., typically with an error of 6%, exceptionally with an error of 12%. Additionally, it was shown that TRM can extract the thickness of SUT even for sheets that are a few times thinner than the illumination wavelength.

## Figures and Tables

**Table 1:** Experimental results for single-layer structures with compensating error sources in TRM.

	Error Mitigation	PVC (102.83 mm)	PVC (50.47 mm)	PVC (20.11 mm)	PVC (9.84 mm)	Vubonite (5.84 mm) [32]
Number of measurements	—	5	4	6	6	8
Time interval point to point (ps)	—	8	4	2	1	0.4
Thickness (mm) (Caliper)	—	102.83±0.04	50.47±0.02	20.11±0.03	9.84±0.03	5.84±0.06
Thickness of sample (mm)	A0	104.04±0.04	52.70±0.03	22.01±0.04	10.17±0.06	6.14±0.03
Thickness of sample (mm)	A1	103.00±0.05	52.00±0.03	21.82±0.05	10.08±0.04	6.07±0.03
Relative error for thickness	—	Less than 0.5%	3.0%	8.0%	2.0%	4.0%
$A_{\left(t_{0}\right)}$ (normalized)	—	1.00	1.00	1.00	1.00	1.00
$PP_{\left(t_{0}\right)}$ → $\Gamma_{01}$(front side)	—	−0.26 + 0.01j	−0.26 + 0.01j	−0.26 + 0.01j	−0.26 + 0.01j	−0.31 + 0.14j
$\varepsilon_{r}\left(\mathrm{SUT}\right)$	A0	2.90 − 0.12j	2.90 − 0.12j	2.90 − 0.12j	2.90 − 0.12j	2.86 − 2.02j
$\varepsilon_{r}\left(\mathrm{SUT}\right)$	A1	2.93 − 0.13j	2.93 − 0.13j	2.93 − 0.13j	2.93 − 0.13j	2.90 − 2.05j
$PP_{\left(t_{0}+t_{1}\right)}$*ε* → $\Gamma_{10}$(back side)	—	0.17 − 0.00j	0.27 − 0.02j	0.28 − 0.01j	0.27 + 0.01j	0.30 − 0.12j
$A_{\left(t_{0}+t_{1}\right)}^{+}$ (normalized)	—	0.68	1.07	1.08	1.05	1.03
$\Gamma_{10}$(back side) = $\frac{V^{-}}{V^{+}}$ →	—	0.25 − 0.00j	0.25 − 0.02j	0.26 − 0.01j	0.26 + 0.01j	0.29 − 0.12j
$\varepsilon_{r}\left(\mathrm{air}\right)$ behind sample	A0, D0	1.46 − 0.06j	0.96 + 0.04j	0.92 − 0.00j	0.96 − 0.00j	1.03 − 0.09j
$\varepsilon_{r}\left(\mathrm{air}\right)$ behind sample	A0, D1	1.04 − 0.04j	1.04 + 0.06j	1.00 − 0.00j	1.00 − 0.00j	1.07 − 0.10j
$\varepsilon_{r}\left(\mathrm{air}\right)$ behind sample	A1, D0	1.30 − 0.06j	0.96 + 0.04j	0.92 + 0.00j	0.96 − 0.00j	1.03 − 0.09j
$\varepsilon_{r}\left(\mathrm{air}\right)$ behind sample	A1, D1	1.04 − 0.05j	1.04 + 0.05j	1.00 + 0.00j	1.00 − 0.00j	1.08 + 0.10j
Relative error for $\varepsilon_{r}\left(\mathrm{air}\right)$	—	6%	6%	<0.5%	<0.5%	12%
